# Genetic Diversity of Pineapple (*Ananas comosus*) Germplasm in Malaysia Using Simple Sequence Repeat (SSR) Markers

**DOI:** 10.21315/tlsr2020.31.3.2

**Published:** 2020-10-15

**Authors:** Siti Norhayati Ismail, Nurul Shamimi Abdul Ghani, Shahril Firdaus Ab. Razak, Rabiatul Adawiah Zainal Abidin, Muhammad Fairuz Mohd Yusof, Mohd Nizam Zubir, Rozlaily Zainol

**Affiliations:** 1 Biotechnology and Nanotechnology Research Centre, Malaysian Agriculture Research and Development Institute (MARDI) Headquarters, Persiaran MARDI-UPM, 43400 Serdang, Selangor, Malaysia; 2 Horticulture Research Centre, MARDI Pontian, KM 53, Jalan Johor, 82000 Pontian, Johor, Malaysia; 3 Industrial Crop Research Centre, Malaysian Agricultural Research and Development Institute (MARDI) Headquarters, Persiaran MARDI-UPM, 43400 Serdang, Selangor, Malaysia

**Keywords:** Molecular Marker, Co-dominant, Simple Sequence Repeat (SSR), Germplasm Characterisation, Penanda Molekul, Kodominan, *Simple Sequence Repeat* (SSR), Pencirian Janaplasma

## Abstract

Assessments of genetic diversity have been claimed to be significantly efficient in utilising and managing resources of genetic for breeding programme. In this study, variations in genetic were observed in 65 pineapple accessions gathered from germplasm available at Malaysian Agriculture Research and Development Institute (MARDI) located in Pontian, Johor via 15 markers of simple sequence repeat (SSR). The results showed that 59 alleles appeared to range from 2.0 to 6.0 alleles with a mean of 3.9 alleles per locus, thus displaying polymorphism for all samples at a moderate level. Furthermore, the values of polymorphic information content (PIC) had been found to range between 0.104 (TsuAC035) and 0.697 (Acom_9.9), thus averaging at the value of 0.433. In addition, the expected and the observed heterozygosity of each locus seemed to vary within the ranges of 0.033 to 0.712, and from 0.033 to 0.885, along with the average values of 0.437 and 0.511, respectively. The population structure analysis via method of delta K (ΔK), along with mean of L (K) method, revealed that individuals from the germplasm could be divided into two major clusters based on genetics (K = 2), namely Group 1 and Group 2. As such, five accessions (Yankee, SRK Chalok, SCK Giant India, SC KEW5 India and SC1 Thailand) were clustered in Group 1, while the rest were clustered in Group 2. These outcomes were also supported by the dendrogram, which had been generated through the technique of unweighted pair group with arithmetic mean (UPGMA). These analyses appear to be helpful amongst breeders to maintain and to manage their collections of germplasm. Besides, the data gathered in this study can be useful for breeders to exploit the area of genetic diversity in estimating the level of heterosis.

## Abstract (ms)

Penilaian ke atas diversiti genetik adalah penting bagi penggunaan dan pengurusan sumber genetik yang efisien dalam program pembaikbakaan. Kepelbagaian genetik dapat diperhatikan pada 65 aksesi nanas yang dikumpulkan daripada koleksi janaplasma MARDI yang berada di Pontian, Johor dengan menggunakan 15 penanda *simple sequence repeat* (SSR). Keputusan menunjukkan sejumlah 59 alel antara 2 hingga 6 dengan purata sebanyak 3.93 alel bagi setiap lokus, dan ini menunjukkan tahap polimorfisma yang sederhana bagi seluruh individu. Selain itu, nilai kandungan maklumat polimorfisma (PIC) yang ditemui adalah antara 0.104 (TsuAC035) hingga 0.697 (Acom_9.9) dengan jumlah purata sebanyak 0.433. Tambahan pula, keheterozigotan yang dijangka dan diperhatikan adalah berbeza antara 0.033 hingga 0.885 dan 0.033 hingga 0.712 dengan purata masing-masing antara 0.511 dan 0.437. Analisa struktur populasi menggunakan kaedah delta K (ΔK) serta kaedah purata L (K) menunjukkan bahawa individu daripada janaplasma nanas ini dapat dibahagikan kepada dua kumpulan genetik utama (K = 2) yang diberi nama Kumpulan 1 dan Kumpulan 2. Lima aksesi (Yankee, SRK Chalok, SCK Giant India, SC KEW5 India dan SC1 Thailand) telah dikumpulkan di dalam Kumpulan 1 manakala yang selebihnya di dalam Kumpulan 2. Penemuan ini turut disokong oleh dendrogram yang dibina menggunakan kaedah *unweighted pair group with arithmetic mean* (UPGMA). Analisa ini sangat membantu pembiakbaka dalam mengekalkan dan mengurus koleksi janaplasma mereka. Di samping itu, data-data yang dikumpulkan dalam kajian ini sangat berguna kepada pembiakbaka dalam mengeksploitasikan diversiti genetik bagi menganggar tahap heterosis.

**Highlights**

- Genetic diversity of 65 pineapple accessions from MARDI’s germplasm had been assessed by using 15 polymorphic SSR markers where moderate level of variations was observed.
- Two major clusters that separated the 65 accessions into two major groups were identified.
- All accessions can be differentiated from one another except for SG Spinal local and LT_SG that had a very low genetic distance.

## INTRODUCTION

Homonym and synonym are common nomenclatures that refer to pineapples (*Ananas comosus* (L.) Merr.), primarily due to the variations of naming customs among breeders and cultivators from within or between nations (Vanijajiva 2012; Feng *et al*. 2013). These have led to redundancy and major mix-up, which have subsequently caused numerous problems that involve genetic resource utilisation and management in breeding programme. Hence, plant genetic resources (PGR), such as collection of germplasm, seems to be some of the significant aspects for enhancement of crop varietal and agrobiodiversity. In fact, based on International Plant Genetic Resource Institute (IPGRI, 1993), PGR is comprised of modern and obsolete cultivars; weeds and wild species; genetic stocks and breeding lines; as well as landraces and cultivated plant species from the primitive line. Moreover, breeders of plants carry out their programmes by depending on the availability of genetic variability, which can be found in their collection of germplasm. Information pertaining to the characterisation of morphological and agronomic features is essential in facilitating use of germplasm among breeders. Besides, characterisation of germplasm refers to the process of recording distinct and unique features, hence suggesting heritable probability (Upadhyaya *et al*. 2008). Most plant breeders depend solely on morphological data (traditional method) to identify and to characterise an accession, which is time-consuming and tedious. In fact, plant morphology can also be affected by environmental conditions, thus leading to inaccuracy.

To date, molecular marker technique appears to be a method of choice for various applications due to its rapidness, accuracy and robust outcomes, hence, displaying great reliability to assist and to support the traditional method. Pervaiz *et al*. (2010) has demonstrated the application of simple sequence repeat (SSR) markers in genetic diversity study on Pakistani rice landraces where four major clusters were generated that can distinguished between the tall, late maturing and slender aromatic types to three other types which were the short, the early and the bold non-aromatic types. In addition, Randhawa *et al*. (2013) discussed the application of molecular markers in marker-assisted breeding (MAB) for disease resistance in wheat. On top of that, molecular markers were also applied in classifying gastric cancer (Hu *et al*. 2012), as well as for fisheries management and conservation (Carvalho & Pitcher 2012).

Therefore, exploration of genetic diversity, as well as its correlations amongst the various accessions can assist plant breeders in their breeding programme, especially in estimating heterosis and hybrid vigour. Genetic diversity research works, particularly on pineapple, were carried out in the past by employing some types of molecular markers. Assessments of genetic diversity and the relationship of Thailand’s pineapple cultivar via inter-simple sequence repeat (ISSR) markers revealed three clusters that distinguished Spanish, Queen, and Cayenne groups (Vanijajiva 2012). On the other hand, genetic diversity at low level was noted for the collection of Cuban pineapples through the use of Amplified Fragment Length Polymorphism (AFLP) markers (Paz *et al*. 2012). A prior study reported using the SSR to look into a genetically diverse pineapple breed in Japan (Shoda *et al*. 2012). Meanwhile, another study determined ten SSR primers (originally designed from *A. bracteatus* genome) that amplified *A. comosus* genome when the SSR actually reflects species-specific that conserves sequences across various species of the same family (Rodríguez *et al*. 2013). This technology of SSR marker was chosen in this study, mainly because of its co-dominant nature, high abundance across genome, applicable to high-throughput technology, and highly polymorphic due to its multi-allelic attribute (Kalia *et al*. 2011; Zhang *et al*. 2011; Shoda *et al*. 2012; Zhang *et al*. 2012).

The description of plant accessions in a systematic manner may suggest sectors that are organised in an excellent shape, thus enhancing and facilitating plant germplasm usage. Hence, this study assessed the genetic diversity of 65 pineapple accessions gathered from germplasm available at Malaysian Agriculture Research and Development Institute (MARDI), which were collected from many locations, including all South East Asian nations, Australia, and Pakistan.

## MATERIALS AND METHODS

### Plant Materials and Molecular Markers

A total of 10 plants from 65 accessions were collected from MARDI’s pineapple germplasm in Pontian, Johor, in order to gather their young leaves. The collected plants had been grown in peat soil, which were taken care of by employing standard cultural practices (no hormone is used to induce flowers), as recommended by Fruit Programme established at the Horticulture Research Centre of MARDI. In the beginning, 56 SSR markers that relatively yielded a high number of alleles and heterozygosity were opted for optimization (Kinsuat & Kumar 2007; Wöhrmann & Weising 2011; Shoda *et al*. 2012).

### DNA Extraction, Quality Assessment and Normalisation

A modified high-throughput plant DNA extraction technique was employed to extract total genomic DNA from individual plants (Zhanguo & Junping 2012). Firstly, a tungsten bead (3 mm) was added to each of the 96 wells with 1 mL round bottom assay block (Corning Incorporated, USA). Next, 0.5 mg of fresh pineapple leaf was added into the wells and incubated overnight in a freezer (−80°C). After that, all the frozen leaf samples inside the plate were ground (30 Hz) for a minute using Tissue Lyser (Qiagen, Netherlands). Later, 600 μL of DNA extraction buffer containing 200 mM of EDTA, 0.1% of *β* mercaptoethanol, 2% of CTAB, 1.2 M of NaCl and 100 mM of Tris (pH 8.0) was added to the plate wells by using a multichannel pipette and mixed well before the samples were incubated for an hour in 60°C water bath (Memmert, German). After the plate was cooled down for 5 min, a ratio of 350 μL chloroform: isoamyl alcohol (24:1) was included into each well, which was then mixed and centrifuged (Beckman Coulter, USA) for 15 min at 5500 rpm. In addition, an equal volume of cold isopropanol was applied for DNA precipitation and then, it was washed using 70% ethanol. Finally, the produced pellets of DNA were air-dried and re-suspended in 50 μL of Tris-EDTA (TE) buffer. The gel electrophoresis was carried out by using 0.8% ethidium bromide pre-stained agarose gel, and then, further visualised under ultraviolet light using Quantum ST4 3000 (Thermo Scientific, USA), in order to determine the quality of the extracted DNA. After that, the Thermo Labsystems Floroskan Ascent™ (Thermo Scientific, USA) was used to determine the extracted DNA’s concentration, which was later normalised to 30 ng μL^−1^.

### PCR Amplification and Fragment Analysis

A total reaction of 10 μL was utilised to carry out polymerase chain reaction (PCR) by using a thermal cycler (Applied Biosystems, USA). The PCR mixture consisted of 10X PCR buffer (Invitrogen™, USA), 50 mM MgCl2 (Invitrogen™, USA), 2 mM dNTPs (Invitrogen™, USA), 10 μM of forward primer anchored with M13 tail (Schuelke 2000), 10 μM of unlabelled reverse primer and 5 μM of M13 tail (fluorescently dyed with FAM, VIC, PET or NED), 5U Taq Polymerase (Invitrogen™, USA), and 30 ng μL^−1^ of DNA template. The thermal conditions of the PCR were set to: initial denaturation for 5 min at 95°C, next followed by 30 cycles (1 min at 95°C, 1 min at respective annealing temperature for every primer pair (see Table 1), then an extension of a minute at 72°C) and lastly, the last extension carried out for 10 min at 72°C. The fragment analysis had been carried out by using a DNA Analyzer (ABI3730XL, Applied Biosystems, USA) with GeneScan™ 500 LIZ (Applied Biosystems, USA) as the size standard to detect DNA fragments ranging from 35 to 500 base pair (bp).

### Statistical Analysis

The DNA fragments produced by DNA analyser was determined by employing the GeneMapper Software 4.0 (Applied Biosystems, USA). This genotypic data were then converted into several different formats by using CONVERT software (Glaubitz 2004). Scoring error, as well as the existence of large allele dropout and null alleles, were investigated by applying the Micro-Checker 2.2 (Cock *et al*. 2004), whereas the estimated and recorded heterozygosity, number of alleles (NA) per locus as well as the deviation from Hardy Weinberg Equilibrium (HDW) using chi-square test and likelihood ratio test were calculated by using POPGENE 1.31 (Yeh *et al*. 1999). Next, the Power Marker software 3.5 was employed in order to determine the aspect of polymorphism information content (PIC) for each SSR marker (Liu & Muse 2005). The Bayesian analysis for structure of population was performed via STRUCTURE 2.3.4 (Pritchard *et al*. 2000). Next, the number of clusters (K) was identified via two approaches: (i) ad hoc statistics, ΔK, which depended on the rate of change in the second-order for data of log probability between the determined values of K (Evanno *et al*. 2005), and (ii) a plot of mean likelihood per K value; L (K) (Pritchard *et al*. 2000; Evanno *et al*. 2005). The Table of Evanno to calculate ΔK and the mean of Ln P (K) were extracted by employing the STRUCTURE HARVESTER (http://taylor0.biology.ucla.edu/structureHarvester). Lastly, the UPGMA dendrogram was formulated via Power Marker software 3.5 by calculating the genetic distance (Nei 1983) and subsequently, by employing the MEGA 7.0.18, the outcome was visualised (Kumar *et al*. 2016).

## RESULTS AND DISCUSSIONS

Initially, 25 out of 56 SSRs displayed exceptional amplifications after optimisation. Meanwhile, the rest were discarded due to non-specific binding and poor amplification in 2% pre-stained (ethidium bromide) agarose gel electrophoresis. As a result, 15 polymorphic SSRs with good amplification’s quality had been selected and used for further analysis across the 65 accessions of pineapple germplasm, while the others were disregarded due to monomorphic alleles and poor amplification during fragment analysis. More information pertaining to each SSR marker is presented in Table 1.

Evidently, the scoring error, the large allele dropout, and null alleles were not recorded for all the 15 SSR markers after they were analysed via Micro- Checker 2.2 hence the population is probably in Hardy Weinberg equilibrium (HWB). Nevertheless, 59 alleles had been identified with a mean of 3.93 alleles per locus at the range of 2.0 to 6.0 alleles. In comparison, Lin *et al*. (2015) had screened 27 pineapple cultivars using 16 SSR markers and obtained 51 alleles with an average of 3.19 alleles per locus also range from 2.0 to 6.0 alleles while Rodríguez *et al*. (2013) had evaluated six pineapple cultivars using 10 SSR markers and prevailed 26 alleles, ranged from 2.0 to 4.0 alleles with an average of 2.60 alleles per locus. From the three studies, there were not much different in the range of alleles observed for each locus (2.0–6.0 alleles), even though the number of cultivar representing low (6), medium (27) and high numbers (65) and the number of alleles ranged from 26 to 59 alleles. This observation may suggest that the variations within the SSR locus in pineapple cultivars are low.

Meanwhile, the pairwise genetic distances appeared to range from 0.0014 until 0.4949 and displayed an average of 0.2284 with exclusion of data. MRTxSS194JK and Yankee had the highest genetic distance (0.4949), while SG Spinal Local and LT_SG had the lowest genetic distance (0.0014). However, the lowest genetic distance is suspected due to missing alleles therefore SG Spinal Local and LT_SG cannot be differentiated between one another or both could possibly be from the same accession. Hence, these data can also be used to identify redundant accessions or varieties as this is one of the major concerns in pineapple plantations. Vegetative plant materials can easily be exchanged from one region to another. Due to non-standard nomenclatures in pineapple, the accessions can easily mix up amongst one another and subsequently cause homonym and synonym. Accessions that have the value of genetic distances below 0.1000 are highly likely suspected to be of the same accessions. However, more markers will be tested on these accessions to verify it in the future. Morphological evaluations will also be conducted to certify the accession. Additionally, the values of PIC seemed to range from 0.1040 (TsuAC035) until 0.6970 (Acom_9.9) at the average of 0.4330. Referring to Rekha *et al*. (2016), the variations of the studied population is in moderate level, since the average of PIC value is in between 0.2500 to 0.5000. Furthermore, the estimated and the recorded heterozygosity appeared to vary from 0.0330 until 0.7120 and 0.0330 until 0.8850 along with averages of 0.4370 and 0.5110, respectively. These data also imply a moderate level of variations in the population. In addition, chi-square tests and likelihood ratio test showed that most of the locus are in agreement with HWB with the exception of TsuAC035 where the probability value is higher than 0.05. HWB law states that frequencies of allele and genotype for a population will reside uniform from one generation to another with exclusion of evolutionary influences such as inbreeding, selection, mutation, genetic drift and gene flow.

**Figure 1:**
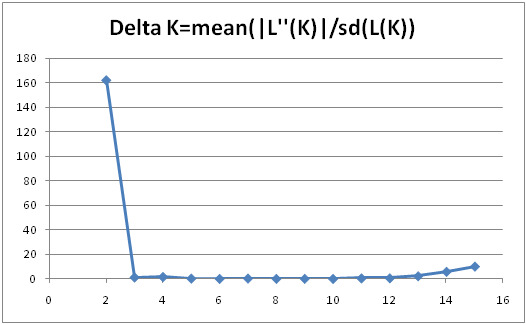
Scatter plot to determine true K value using log probability (ΔK) method; the highest value of ΔK represents the optimum K value (K = 2) (Evanno *et al*. 2005).

**Figure 2:**
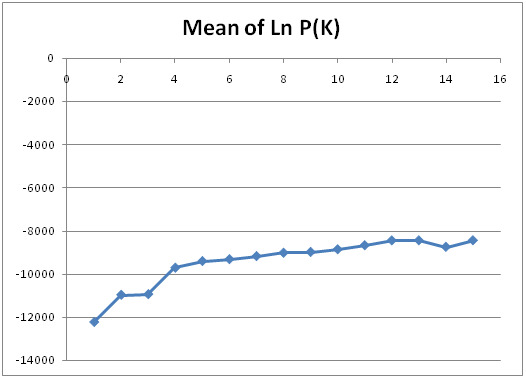
Determination of K value using mean of Ln P (K) method; Ln P (K) plateaus and the variation between runs increases when approaching an optimum K value (Pritchard *et al*. 2000; Evanno *et al*. 2005). K = 2 shows the first plateau with the highest variations.

**Figure 3:**
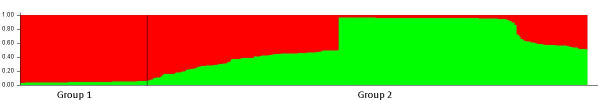
Bar plot structure of K = 2 obtained by STRUCTURE software version 2.3.4. The plot shows two major clusters separated by a straight line (Group 1 and Group 2).

The analysis exhibited that after a total of 10 runs for every K value (1–10), along with burn-in from 10,000 until 100,000 iterations; the optimal K value was determined as 2 (K = 2), thus implying that individuals from the germplasm can be segregated into two major clusters of genetic. Besides, the Table of Evanno displays the values of ΔK for every K (1–10), whereby the optimal number of K is represented by the highest yield of ΔK (K = 2), as given in Table 2. A scatter plot was constructed based on the value of ΔK which shows 2 as the optimal number of K (Fig. 1). Moreover, the population structure analysis,which had been determined via mean likelihoods per K value and Ln P (K) method also suggested K = 2 (Fig. 2). The Ln P (K) plateaus and the variation between runs seemed to escalate upon approaching the optimum K value. In addition, K = 2 portrays the first plateau with the highest variations, thus indicating the K optimum value (Pritchard *et al*. 2000; Evanno *et al*. 2005). The structure pattern for K = 2 is presented in the bar plot, where the two major clusters (Groups 1 and 2) were divided by a straight line (see Fig. 3). Unfortunately, distinctive morphological characteristic between clusters cannot be determined as pineapple breeders in MARDI still in the process to evaluate the germplasm collection. Furthermore, Lin *et al*. (2015) also revealed that based on the study of 64 accessions using 57 SNP markers, two clusters (K = 2) was the most probable number of K where all accessions related to var. *bracteatus* and *erestifolius* were clustered together in one group while the other accessions from var. *ananassoides* was clustered in another group.

**Figure 4:**
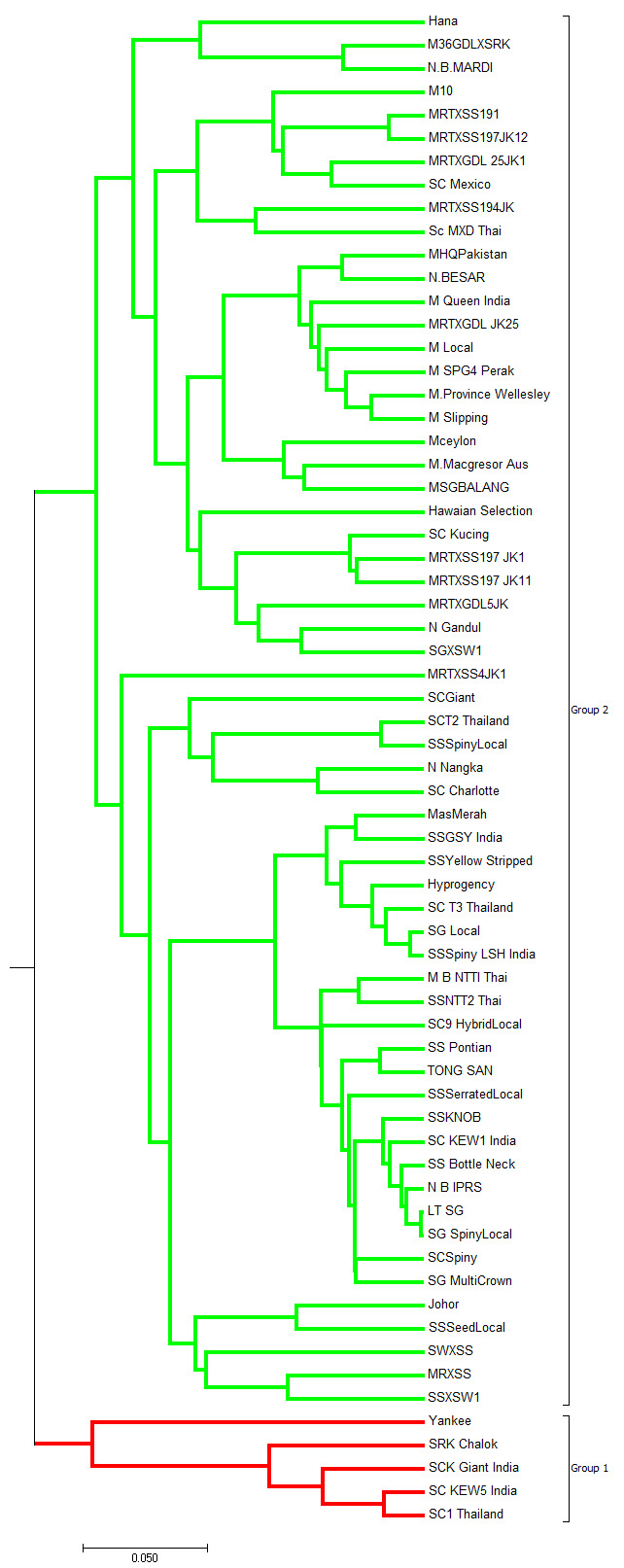
UPGMA cluster dendrogram showing the relationships of 65 pineapple accessions based on 15 polymorphic SSR markers. Two major clusters can be observed (Group 1 and Group 2).

These observations are in line with the results obtained from dendrogram via UPGMA analysis, where the two major clusters (Groups 1 and 2) had been noted (see Fig. 4). In fact, five accessions (Yankee, SRK Chalok, SCK Giant India, SC KEW5 India and SC1 Thailand) were clustered in Group 1, while the rest were clustered in Group 2.

## CONCLUSION

This study reveals that MARDI’s pineapple germplasm has a moderate range of diversity. In fact, the data retrieved appear useful for plant breeders, especially in estimating the level of heterosis, where the offspring of the two breed lines carried superior traits or characteristics, in comparison to both of their parents. Furthermore, it was noted that the higher the diversity between two breed lines, the higher the chances of heterosis. In addition, the outcome of this study can be applied as a significant tool for breeders to maintain and to manage their germplasm collections systematically and efficiently.

## Figures and Tables

**Table 1:** Diversity information parameters on the 15 polymorphic SSR markers.

Locus	Repeat motive	Ta^a^	Na^b^	H_o_^c^	H_e_^d^	PIC^e^
TsuAC004	(AG)16	64	4	0.885	0.634	0.618
TsuAC008	(GA)16	56	5	0.611	0.483	0.504
TsuAC010	(GT)14A(AG)12	53	5	0.266	0.335	0.401
TsuAC013	(AGAGAT)3(AG)12	66	5	0.819	0.527	0.452
TsuAC018	(CA)10A(AC)9	65	3	0.277	0.500	0.415
TsuAC023	(CA)10(TA)11	66	3	0.346	0.417	0.383
TsuAC030	(AG)27	51	4	0.744	0.504	0.488
TsuAC035	(GA)9	62	2	0.033	0.033	0.104
ACPCT136B	(GAC)7	62	5	0.737	0.712	0.675
Acom_9.9	(TTC)8	62	6	0.792	0.666	0.697
Acom_22.22	(AAG)6	62	3	0.198	0.182	0.289
Acom_39.5	(TGG)5	65	3	0.151	0.162	0.220
Acom 65.1	(AGT)5	47	2	0.391	0.500	0.424
Acom 68.3	(AT)8	55	3	0.549	0.398	0.395
Acom 82.8	(GT)10	62	3	0.868	0.500	0.433
Mean			3.733	0.511	0.437	0.433

*Notes:* Ta^a^ = annealing temperature; Na^b^ = Number of alleles; H_o_^c^ = observed heterozygosity; H_e_^d^ = expected heterozygosity and PIC^e^ = polymorphic information content.

**Table 2:** Evanno table output generated by STRUCTURE HARVESTER.

K	Reps	Mean LnP(K)	Stdev LnP(K)	Ln′(K)	|Ln″(K)|	Delta K
1	11	−11480.1273	0.179393	–	–	–
**2**	**11**	**−10197.4909**	**2.373375**	**1282.63636**	**815.80909**	**343.73375**
3	11	−9730.66364	248.356881	466.827273	170.78182	0.687647
4	11	−9093.05455	120.521412	637.609091	687.71818	5.706191
5	11	−9143.16364	277.076478	−50.109091	58.754545	0.212052
6	11	−9134.51818	1028.3617	8.645455	26.181818	0.02546
7	11	−9152.05455	1015.05906	−17.536364	778.15455	0.76661
8	11	−8391.43636	237.502052	760.618182	695.46364	2.928243
9	11	−8326.28182	366.903981	65.154545	109.21818	0.297675
10	11	−8151.90909	96.825074	174.372727	–	–

*Note*: Data in bold is the highest value for Delta K.
